# Impact of the COVID-19 Pandemic on Stage at Presentation Among Breast Cancer Patients: A Multicenter Retrospective Study

**DOI:** 10.7759/cureus.90799

**Published:** 2025-08-23

**Authors:** Faisal Nabi Depar, Marium Nadeem Khan, Sanam Nusrat, Naveed Uddin, Muhammad Imran Farid, Mohammad Ibrahim Rasool, Ayesha Inam, Hafiz Ali Raza

**Affiliations:** 1 Department of Medicine, People’s University of Medical and Health Sciences for Women, Nawabshah, PAK; 2 Department of Medicine, Shifa International Hospitals Limited, Islamabad, PAK; 3 Department of General Surgery, Mediclinic Middle East, Dubai, ARE; 4 Department of Internal Medicine, King Fahd Armed Forces Hospital, Jeddah, SAU; 5 Department of Electrical and Computer Engineering, Air University, Islamabad, PAK; 6 Department of Surgery, Naseer Teaching Hospital/Kabir Medical College, Peshawar, PAK; 7 Department of Agriculture Extension, Muhammad Nawaz Shareef University of Agriculture, Multan, PAK

**Keywords:** breast cancer, covid-19, delayed diagnosis, pakistan, pandemic impact, stage at diagnosis

## Abstract

Background

Global healthcare systems were upended by the COVID-19 pandemic, which resulted in delayed cancer diagnosis and possible changes to disease staging at presentation.

Objective

The main objective of this study is to assess and compare the stage at which breast cancer patients presented before and during the COVID-19 pandemic, with particular attention to any shift toward more advanced stages at the time of diagnosis.

Methodology

This multicenter, retrospective, observational study was conducted at Shifa College of Medicine, Islamabad; Northwest School of Medicine, Peshawar; and Kabir Medical College, Peshawar. Excluding the peak lockdown period, female patients with new primary breast cancer diagnoses were split into two cohorts: pre-pandemic (March 2019-February 2020) and pandemic (September 2020-August 2021). There were 396 cases in total (204 pandemic and 192 pre-pandemic). Clinical, pathological, and temporal data were examined using IBM SPSS Statistics for Windows, Version 25 (Released 2017; IBM Corp., Armonk, NY, USA). Chi-square tests were used, and a p-value of less than 0.05 was deemed statistically significant.

Results

The number of patients receiving advanced-stage (Stage III-IV) diagnoses during the pandemic rose from 72 (37.50%) to 110 (53.92%), with Stage IV cases almost tripling from 17 (8.85%) to 33 (16.18%) (p < 0.05). Between Stage I and II, the number of early-stage patients decreased from 120 (62.50%) to 94 (46.08%). Treatment delays >60 days increased from 21 (10.94%) to 53 (25.78%), and diagnostic delays >60 days were more prevalent during the pandemic (75 patients, 36.76%) than before (51 patients, 26.56%). The average duration between diagnosis and therapy increased from 29.7 ± 14.8 to 42.3 ± 19.6 days, and from symptom onset to diagnosis, it increased from 47.6 ± 18.3 to 61.2 ± 21.5 days (p < 0.05).

Conclusion

The COVID-19 pandemic led to delayed diagnosis and a significant increase in advanced-stage breast cancer presentations in Pakistani women.

## Introduction

The late 2019 onset of the COVID-19 pandemic caused severe disruptions to healthcare systems worldwide, diverting resources to urgent pandemic-related care and significantly impacting the management of cancer and other chronic illnesses [1-3]. As the most prevalent cancer among women globally, breast cancer outcomes rely heavily on timely detection, diagnosis, and treatment [3,4]. The abrupt and prolonged disruption of routine medical services, such as elective diagnostic procedures and mammography screening programs, raised serious concerns about delayed cancer identification and the potential for worsened prognoses [5].

In Pakistan, as elsewhere, the pandemic did not unfold as a single, uniform phase. Between December 2019 and March 2020, most coronavirus cases were confined to China; however, Pakistan reported its first confirmed cases in late February 2020, followed by a nationwide lockdown in March [2,6]. During this period, outpatient services and elective procedures were largely suspended, while subsequent phases saw partial reopening under strict restrictions, such as curfews, travel limitations, and distancing measures [7]. These challenges were further compounded by long-standing socioeconomic constraints, transportation barriers, and limited health awareness, which also contributed to delays in timely breast cancer diagnosis in Pakistan [8-10].

As a result of these disruptions, many patients postponed or cancelled medical visits during the peak of the pandemic, whether due to system-level barriers or fear of virus exposure [6,7]. In the context of breast cancer, such delays are particularly critical, as early detection is strongly associated with lower-stage disease, less intensive treatment, and improved survival [8-12]. After the onset of the pandemic, patients increasingly presented with more advanced illness - a trend likely linked to interruptions in healthcare delivery and delays in seeking medical attention [13].

Several international studies have suggested a possible reversal of decades of progress in early detection, showing increases in tumor size, lymph node involvement, and advanced staging among patients diagnosed during the pandemic [14]. These shifts not only affect individual prognoses but also place additional strain on public health systems, which may be forced to manage more complex cases with greater resource demands. To strengthen system resilience and mitigate long-term consequences, it is essential to assess the specific impact of the pandemic on breast cancer presentation within local contexts. This study aims to assess and compare the stage at which breast cancer patients presented before and during the COVID-19 pandemic in Pakistan, with particular attention to shifts toward more advanced stages at diagnosis. This multicenter study, conducted across three tertiary care hospitals, also situates findings within the unique context of the pandemic in Pakistan, where strict quarantine measures, healthcare service disruptions, and societal adjustments influenced access to timely diagnosis and treatment.

## Materials and methods

Study design and setting

This multicenter, retrospective observational study was conducted at three tertiary care hospitals: Shifa College of Medicine, Islamabad; Northwest School of Medicine, Peshawar; and Kabir Medical College, Peshawar.

Study duration

The study was divided into two phases: a pre-pandemic group (March 2019 to February 2020) and a pandemic group (September 2020 to August 2021), covering a total of two years. The interval from March to August 2020 was deliberately excluded because it coincided with the first wave of COVID-19 in Pakistan and the strictest nationwide lockdowns, during which outpatient clinics, elective surgeries, and screening programs were suspended and access to hospitals was severely restricted. Including this period could have introduced bias, as very few new breast cancer cases were formally diagnosed or recorded.

The pre-pandemic phase represents a baseline period when healthcare services were fully operational. The pandemic phase reflects the “new normal,” when services had resumed but continued under pandemic-related restrictions, such as reduced outpatient volumes, precautionary measures, and patient hesitancy to seek hospital care. These two phases were therefore chosen to allow for a meaningful comparison of breast cancer presentation before and during the pandemic in Pakistan.

Inclusion and exclusion criteria

The study included female patients with new primary breast cancer diagnoses during the defined study periods, who had complete clinical, pathological, and staging data. Male patients, cases with inadequate or missing data on key variables, and patients with recurrent or previously treated breast cancer were excluded.

Sample size and sampling technique

A total of 396 female breast cancer patients were included from the three hospitals. All eligible and complete cases that met the inclusion and exclusion criteria during the study periods were captured across the centers, ensuring comprehensive representation of both pre-pandemic and pandemic phases, and minimizing selection bias. Given the retrospective design, no a priori sample size calculation was performed. Instead, the study aimed to include the entire available cohort of new breast cancer cases with complete records during the defined periods, reflecting real-world clinical patterns of disease presentation. Approximately 132 patients were included from each hospital, and the final sample size is consistent with comparable observational studies in oncology [12,13]. These methodological choices and inherent limitations are acknowledged in the discussion.

Data collection

Retrospective data were extracted from institutional cancer registries and hospital records at all participating centers. Data extraction was carried out independently by trained research staff using standardized forms. All entries were cross-checked by a second investigator, and discrepancies were resolved by consensus. Formal inter-rater reliability testing was conducted on a random sample of 50 cases, with Cohen’s kappa coefficients of 0.87 for staging and 0.82 for treatment delay intervals, demonstrating strong agreement. Only cases with complete staging and treatment delay information were included in the final analysis.

The following variables were collected: demographic characteristics (age, residence, socioeconomic background); clinical characteristics (presenting symptoms and comorbidities); tumor features (grade, histological type, and receptor status, including ER, PR, and HER2); and stage at diagnosis, according to the American Joint Committee on Cancer (AJCC 8th edition) [15]. Where available, time intervals were recorded, with diagnostic delay defined as the interval between first patient-reported symptom and histopathological confirmation of breast cancer, and treatment delay defined as the interval between confirmed diagnosis and initiation of first definitive treatment (surgery, chemotherapy, or radiotherapy). Delays were categorized as ≤30 days, 31-60 days, and >60 days.

The use of uniform data collection tools across centers, cross-checking procedures, and standardized definitions ensured accuracy, comparability, and internal validity across sites.

Statistical analysis

Data were analyzed using IBM SPSS Statistics for Windows, Version 25 (Released 2017; IBM Corp., Armonk, NY, USA). Descriptive statistics (mean, standard deviation, frequency, and percentage) were calculated. Comparisons between pre-pandemic and pandemic cohorts were performed using the Chi-square test for categorical variables. A p-value of less than 0.05 was considered statistically significant.

Ethical approval

The study was approved by the Institutional Review Boards (IRBs) of all participating institutions. Patient anonymity and confidentiality were strictly maintained, and all data were used solely for research purposes.

## Results

**Figure 1:**
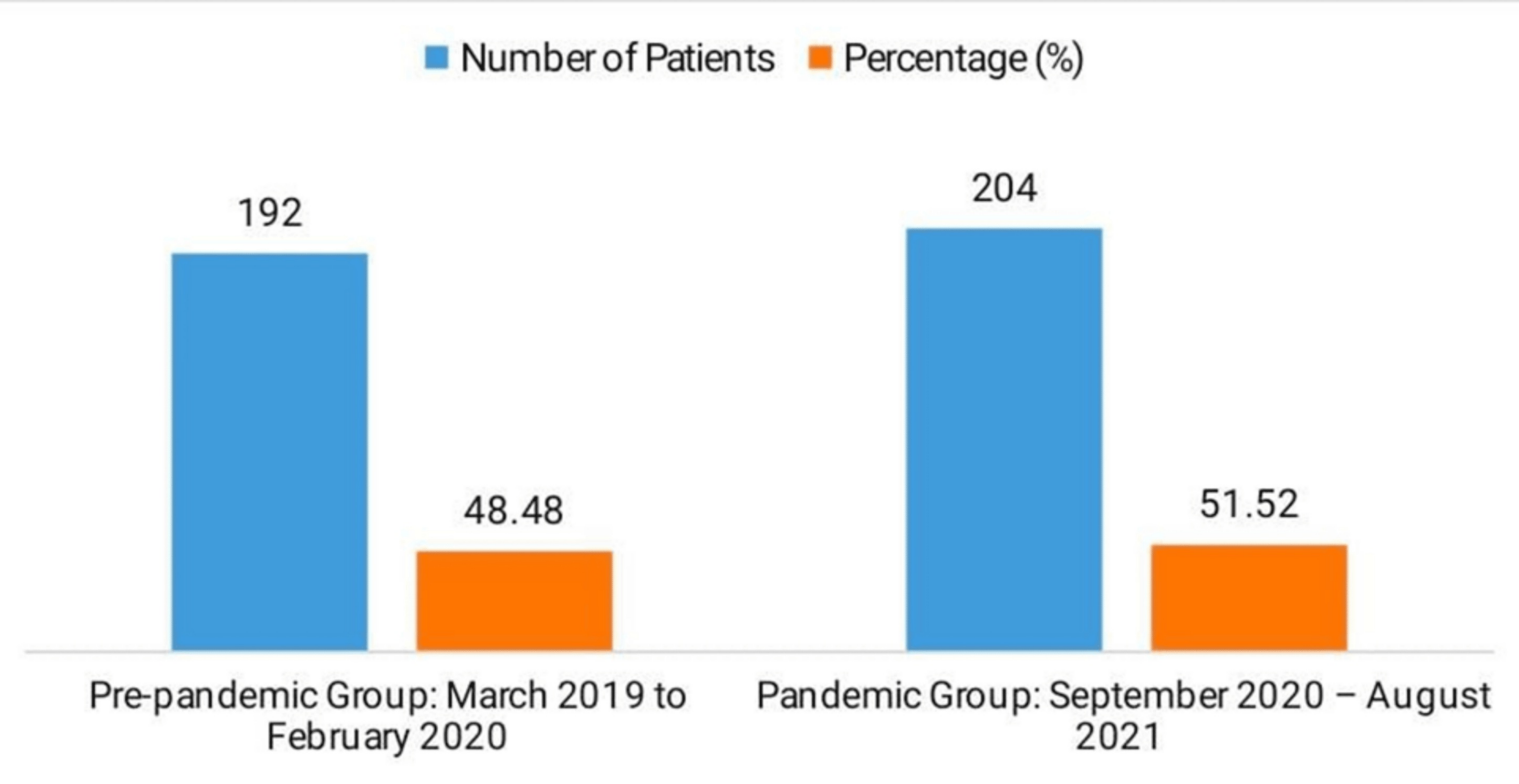
Distribution of Breast Cancer Patients by Study Period

The study included 396 patients with breast cancer, of whom 192 (48.48%) received their diagnosis prior to the pandemic (March 2019 to February 2020), and 204 (51.52%) during the pandemic (September 2020 to August 2021), suggesting a marginally higher number of diagnoses during the pandemic (Figure 1).

The age distribution was the same for both groups, with the largest percentage of patients in the pre-pandemic group (67 patients, 34.90%) and the pandemic group (70 patients, 34.31%) being between the ages of 40 and 49 (Table 1). In terms of residence, 112 patients (58.33%) in the pre-pandemic group, and 119 patients (58.33%) in the pandemic group, were urban dwellers. Low socioeconomic status increased somewhat during the pandemic, with 106 patients (51.96%) experiencing it, compared to 96 patients (50.00%) before it. Breast lumps were the most prevalent presenting symptom, recorded by 171 patients (83.82%) during the pandemic, and 154 patients (80.21%) before it. With 71 patients (36.98%) in the pre-pandemic group and 78 patients (38.24%) in the pandemic group, hypertension was the most common comorbidity. The most common histological type was still invasive ductal carcinoma, which was detected in 170 cases (83.33%) during the pandemic, and 157 individuals (81.77%) before it. These baseline characteristics were comparable across both groups, thereby supporting that subsequent differences in stage at diagnosis and delays are more likely related to pandemic-related disruptions rather than underlying patient or tumor factors.

**Table 1:** Demographic, Clinical, and Pathological Characteristics of Breast Cancer Patients by Study Period

Characteristic	Category	Pre-pandemic (n = 192)	Pandemic (n = 204)
Age Distribution	< 40 years	38 (19.79%)	41 (20.10%)
	40-49 years	67 (34.90%)	70 (34.31%)
	50-59 years	53 (27.60%)	58 (28.43%)
	≥ 60 years	34 (17.71%)	35 (17.16%)
Residence	Urban	112 (58.33%)	119 (58.33%)
	Rural	80 (41.67%)	85 (41.67%)
Socioeconomic Status	Low	96 (50.00%)	106 (51.96%)
	Middle	71 (36.98%)	73 (35.78%)
	High	25 (13.02%)	25 (12.25%)
Presenting Symptoms	Breast lump	154 (80.21%)	171 (83.82%)
	Breast pain	42 (21.88%)	48 (23.53%)
	Nipple discharge	17 (8.85%)	22 (10.78%)
	Skin changes	12 (6.25%)	19 (9.31%)
	Axillary mass	26 (13.54%)	33 (16.18%)
Comorbidities	Hypertension	71 (36.98%)	78 (38.24%)
	Diabetes mellitus	45 (23.44%)	56 (27.45%)
	Cardiovascular disease	18 (9.38%)	22 (10.78%)
	No comorbidity	83 (43.23%)	76 (37.25%)
Histological Type	Invasive ductal carcinoma	157 (81.77%)	170 (83.33%)
	Invasive lobular carcinoma	18 (9.38%)	19 (9.31%)
	Mixed type	11 (5.73%)	10 (4.90%)
	Others	6 (3.13%)	5 (2.45%)

Table 2 shows that 89 patients (43.63%) had high-grade malignancies during the pandemic, compared to 71 patients (36.08%) in the pre-pandemic group. However, this difference was not statistically significant (p = 0.122). Prior to the pandemic, 125 patients (65.10%) had ER-positive tumors, while during the pandemic, 128 patients (62.75%) did. Similarly, 117 patients (60.94%) had PR-positive status before the pandemic, compared to 120 patients (58.82%) during it. Additionally, 54 patients (28.13%) in the pre-pandemic group, and 61 patients (29.90%) in the pandemic group, had HER2-positive malignancies. None of these changes reached statistical significance (p > 0.05 for all markers).

**Table 2:** Tumor Characteristics

Variable	Pre-pandemic (n = 192)	Pandemic (n = 204)	p-value
High tumor grade	71 (36.98%)	89 (43.63%)	0.122
ER-positive	125 (65.10%)	128 (62.75%)	0.636
PR-positive	117 (60.94%)	120 (58.82%)	0.693
HER2-positive	54 (28.13%)	61 (29.90%)	0.708

During the pandemic, there was a noticeable trend toward advanced-stage presentation (Table 3). In the pre-pandemic group, there were 120 patients (62.50%) with early-stage cases (Stage I and II); in the pandemic group, there were 94 individuals (46.08%). On the other hand, the number of patients with advanced-stage cases (Stage III and IV) rose from 72 (37.50%) to 110 (53.92%). Specifically, the proportion of patients presenting with Stage IV disease nearly doubled during the pandemic, increasing from 17 individuals (8.85%) in the pre-pandemic group to 33 patients (16.18%) in the pandemic group, representing a statistically significant shift (p < 0.05).

**Table 3:** Clinical Stage at Diagnosis (AJCC 8th Edition) AJCC 8th Edition [15] AJCC, American Joint Committee on Cancer

Stage	Category	Pre-pandemic (n = 192)	Pandemic (n = 204)	p-value
Stage I	Early Stage	39 (20.31%)	26 (12.75%)	<0.05
Stage II	Early Stage	81 (42.19%)	68 (33.33%)	
Stage III	Advanced Stage	55 (28.65%)	77 (37.75%)	
Stage IV	Advanced Stage	17 (8.85%)	33 (16.18%)	

Significantly lengthier delays were encountered by patients who were diagnosed during the pandemic (Table 4). Compared to 83 patients (43.23%) in the pre-pandemic group, only 62 patients (30.39%) received a diagnosis within 30 days after the onset of symptoms. Furthermore, compared to 51 patients (26.56%) before the pandemic, 75 patients (36.76%) experienced delays longer than 60 days during the pandemic. Before the pandemic, the mean period from symptom onset to diagnosis was 47.6 ± 18.3 days; after the pandemic, it was 61.2 ± 21.5 days. Similarly, during the pandemic, 101 patients (49.51%) received treatment within 30 days of diagnosis, whereas 129 individuals (67.19%) did so prior to the pandemic. The number of patients who had delays greater than 60 days between diagnosis and treatment rose from 21 (10.94%) to 53 (25.78%). Additionally, the average time to start therapy increased from 29.7 ± 14.8 days to 42.3 ± 19.6 days. Every difference was statistically significant (p < 0.05).

**Table 4:** Diagnostic and Treatment Delays

Time Interval	Pre-pandemic (n = 192)	Pandemic (n = 204)	p-value
Symptom onset to diagnosis ≤30 days	83 (43.23%)	62 (30.39%)	<0.05
Symptom onset to diagnosis >60 days	51 (26.56%)	75 (36.76%)	
Mean ± SD (onset to diagnosis, days)	47.6 ± 18.3	61.2 ± 21.5	
Diagnosis to treatment ≤30 days	129 (67.19%)	101 (49.51%)	<0.05
Diagnosis to treatment >60 days	21 (10.94%)	53 (25.98%)	
Mean ± SD (diagnosis to treatment)	29.7 ± 14.8	42.3 ± 19.6	

## Discussion

This multicenter retrospective research aimed to assess and compare the stage at presentation of patients with breast cancer before and during the COVID-19 pandemic, with a particular emphasis on detecting any changes toward more advanced illness at diagnosis. According to our research, the pandemic caused major disruptions to the care pathways for breast cancer, which resulted in greater delays in detection and treatment. During the pandemic, there was a noticeable shift toward presentations with more advanced stages, which aligns with global patterns documented in the literature. The percentage of patients in our cohort diagnosed at an advanced stage (Stage III or IV) increased from 37.50% before the pandemic to 53.92% during it (p < 0.05). Interestingly, Stage IV cases increased from 8.85% to 16.18%, almost doubling. These results are consistent with other research showing that advanced-stage diagnoses (Stage III-IV) increased similarly in Brazil and Italy during the pandemic [14,16].

Another important element that surfaced was the delay in diagnosis. Before the pandemic, the mean period from symptom onset to diagnosis was 47.6 ± 18.3 days; after the pandemic, it was 61.2 ± 21.5 days (p < 0.05). Compared to 43.23% before the pandemic, only 30.39% of patients after the pandemic received a diagnosis within 30 days. This pre-pandemic delay is consistent with prior studies from Pakistan, which have documented mean patient delays ranging from approximately 5 months to over 15 months - largely due to lack of awareness, sociocultural barriers, and reliance on alternative treatments [9,17,18]. Thus, while the baseline delay in our cohort appears shorter than national averages, the additional increase during the pandemic highlights the compounding effect of service disruptions. These findings are in line with international reports from Brazil, the Netherlands, and Turkey, where the COVID-19 pandemic similarly resulted in delayed diagnoses and more advanced disease presentations [19-27].

Additionally, there was a significant delay in the start of treatment. Delays beyond 60 days rose from 10.94% to 25.98%, and the mean duration from diagnosis to treatment increased from 29.7 ± 14.8 days prior to the pandemic to 42.3 ± 19.6 days (p < 0.05). These results are consistent with a prior study, which found that among patients with breast cancer identified during COVID-19, treatment start delays beyond 30 days were linked to stage migration and reduced survival rates [28].

High-grade malignancies were more common during the pandemic (43.63% vs. 36.98%), but receptor status and tumor histology were similar in both groups. The difference was not statistically significant (p = 0.122), but it supports the idea that delays may lead to physiologically more aggressive cancers being discovered later. A higher percentage of high-grade and symptomatic tumors was also observed during the pandemic, according to earlier research [20,29].

Collectively, these results demonstrate the unforeseen oncologic effects of the healthcare interruptions brought on by the pandemic. They underscore the critical need for continuity planning in oncology care, including the integration of alternative consultation models, such as telemedicine, mobile screening units, and decentralized treatment protocols. Implementing such strategies in national healthcare policies could ensure timely diagnosis and uninterrupted treatment of cancer patients during future public health emergencies.

Strengths and limitations

This study has several important strengths. The multicenter design, incorporating three tertiary care facilities from different regions of Pakistan, enhances the representativeness of our sample and improves the generalizability of our findings. Internal validity was reinforced by the application of uniform staging criteria (AJCC 8th edition) [15] and the use of standardized data collection forms across all centers, ensuring consistency and comparability between pre-pandemic and pandemic cohorts. Importantly, by excluding the peak lockdown period (March-August 2020), we minimized confounding from a time when healthcare access was most restricted and very few breast cancer cases were formally diagnosed, thereby allowing for a clearer assessment of real-world diagnostic and treatment patterns.

To reduce bias inherent in retrospective designs, we applied several safeguards. All eligible and complete cases from each center were included to avoid selective inclusion. Standardized data extraction protocols were followed, and only records with complete staging and treatment delay information were analyzed to minimize documentation bias. Uniform data-gathering tools were implemented across all sites to ensure consistency in reporting and strengthen the reliability of results. These measures collectively enhance the robustness of our findings and reduce the likelihood that the observed differences are due to methodological weaknesses rather than true effects of the pandemic.

Nevertheless, certain limitations remain. Selection and documentation bias cannot be fully eliminated in retrospective studies, and some variables - particularly socioeconomic details and patient-reported reasons for delay - were incomplete due to reliance on hospital records. External validity may also be limited by the exclusion of male breast cancer cases and recurrence data. However, these limitations do not compromise the principal outcomes of the study. The clear shift toward advanced-stage presentation and longer diagnostic and treatment delays observed in our cohort is consistent with both national and international evidence, underscoring the reliability and broader relevance of our conclusions.

## Conclusions

The COVID-19 pandemic had a substantial impact on breast cancer detection pathways in Pakistan, with more patients presenting at advanced stages and experiencing longer delays in diagnosis and treatment compared to the pre-pandemic period. While these findings align with international data, they should be interpreted with caution, given the retrospective design and hospital-based sample. Nonetheless, the results highlight the importance of developing resilient healthcare strategies that ensure continuity of cancer care during future public health emergencies.
